# Ventral Spinal Cord Herniation Causing Spinal Intradural Hematoma and Subarachnoid Hemorrhage: A Case Report

**DOI:** 10.7759/cureus.28349

**Published:** 2022-08-24

**Authors:** Shyle H Mehta, Kevin A Shah, Cassidy D Werner, Timothy G White, Sheng-Fu L Lo

**Affiliations:** 1 Neurological Surgery, Northwell Health, Manhasset, USA

**Keywords:** ventral spinal cord herniation, intradural hematoma, subarachnoid hemorrhage, spinal cord hemorrhage, spinal cord herniation

## Abstract

Ventral spinal cord herniation is a rare pathology, caused by a dural defect, that leads to progressive myelopathy. The true prevalence of ventral spinal cord herniation is unknown largely because of underdiagnosis due to its nonspecific symptoms. Though there are theories that attempt to describe how these dural defects are formed, the true causes of these defects are unknown. In this case report, we present a case of a 29-year-old female who had an idiopathic ventral spinal cord herniation causing an intradural hematoma and subarachnoid hemorrhage. This is the first reported case of spinal cord herniation causing hemorrhage.

## Introduction

Ventral spinal cord herniation is rare, generally causes progressive myelopathy, and is often misdiagnosed [1]. Spinal cord herniation as a result of the dural defect was first reported in 1974 by Wortzman et al. [2]. Since that time, more cases have been reported largely due to increased recognition with magnetic resonance imaging (MRI) [3].

Given the nonspecific or absence of symptoms of this disease and its insidious course, the true prevalence of ventral spinal cord herniation is unknown [3]. Several theories describe the formation of a dural defect, but the causes of these defects remain unclear [4]. Physiologic kyphosis of the thoracic spine and the relative immobility of the thoracic spinal cord, which allows cerebrospinal fluid (CSF) pulsations to contribute to the progression of the herniation, are hypothesized to be important factors of disease onset [3,5]. This is likely why spinal cord herniation usually occurs along the ventral dura between T2 and T10 [1].

Once a dural defect is present, the spinal cord deviates towards the defect until it herniates through it [6-10]. This is in part driven by physiologic pulsations of CSF that push the cord [6,8,9]. We present a case report of a 29-year-old female who was found to have ventral spinal cord herniation causing intradural hematoma and associated subarachnoid hemorrhage (SAH). Though many cases of spinal cord herniation have been reported, to the best of our knowledge, there are no reported cases of spinal cord herniation-induced hemorrhage.

## Case presentation

**Figure 1:**
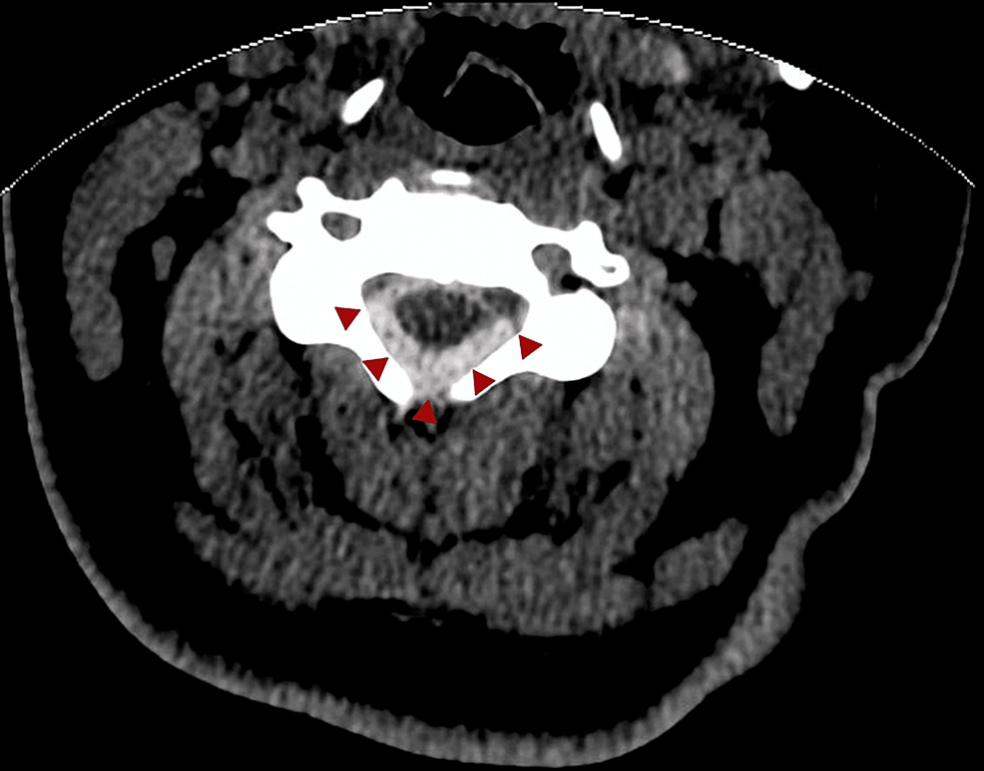
Axial non-contrast computed tomography image of cervical spine demonstrating subarachnoid hemorrhage

**Figure 2:**
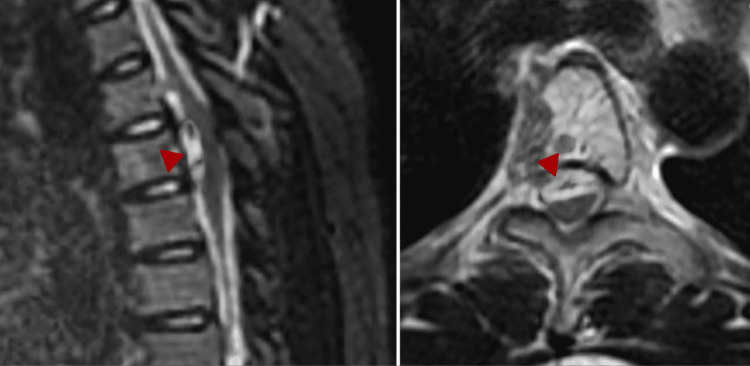
Sagittal (left) and axial (right) T2 magnetic resonance image of thoracic spine demonstrating compressive T4 lesion

A 29-year-old female with a history significant for motor vehicle accident (MVA) 11 months prior presented with acute onset sharp chest pain radiating to her back. At that time, she was taken to a hospital and discharged after her workup was determined to be negative. No spinal imaging was obtained since she was only complaining of chest pain at the time. Due to persistent chest pain and new-onset, progressive lower extremity weakness, the patient went to another hospital where a non-contrast head and spinal CT (Figure 1) were performed. She was subsequently transferred to a third hospital for an emergent spinal MRI (Figure 2). At the time, her neurological exam was significant for markedly reduced strength in her bilateral lower extremities (1/5) and mildly diminished sensation from mid-abdomen down, including saddle anesthesia. Imaging demonstrated cervical spine and cranial SAH as well as a lesion, best seen on T2 sequence, within the ventral spinal canal at T4.

**Figure 3:**
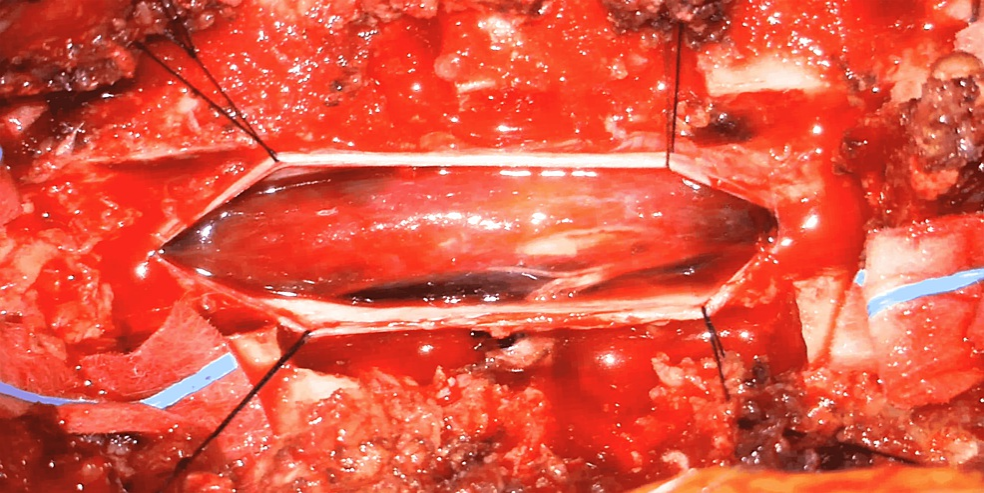
Exposed spinal cord with dura mater retracted and intradural hematoma visualized

**Figure 4:**
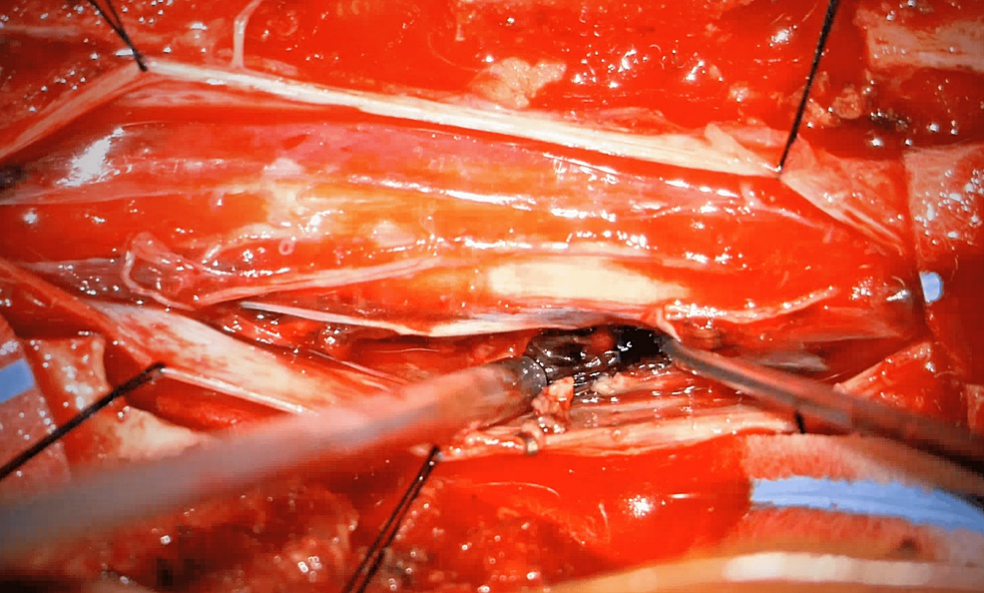
Intradural hematoma being evacuated

**Figure 5:**
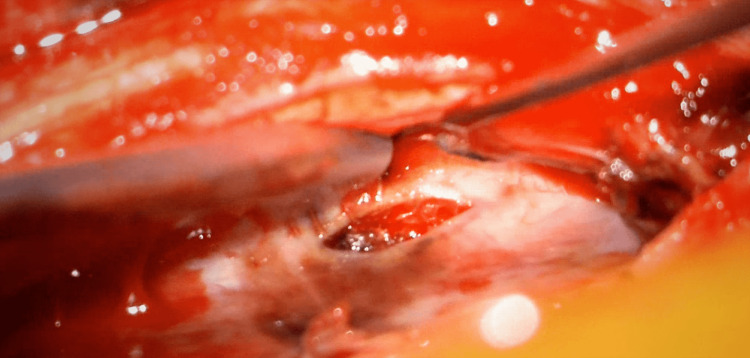
Identification of ventral dural defect once intradural hematoma evacuated

**Figure 6:**
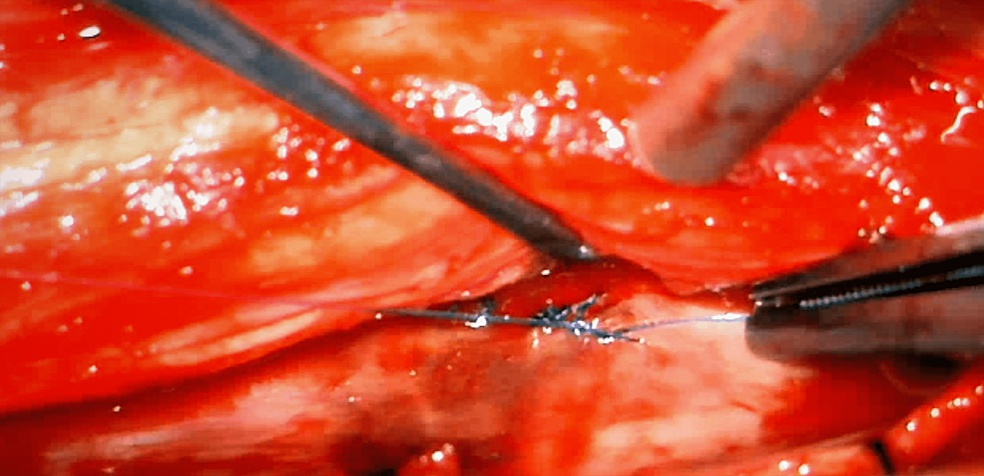
Primary repair of the ventral dural defect

The patient underwent emergent cranial and spinal angiography, which was negative for an underlying vascular malformation. She was then transported directly from the interventional radiology suite to the operating room for a lateral extracavitary approach for exploration and decompression of the T3-T4 lesion. Prior to the procedure, the patient did not have somatosensory evoked potentials (SSEPs) or motor evoked potentials (MEPs). During the procedure, the patient was found to have a large ventral intradural hematoma (Figures 3, 4) overlying a ventral dural defect (Figure 5), which was primarily repaired (Figure 6). A T2-T6 fusion was performed. 

Postoperatively, the patient had a return of SSEPs in bilateral lower extremities. Her exam somewhat improved. After one week, she regained partial motor function in her left foot (2/5 dorsi- and plantar-flexion) and she regained full sensation of her right lower extremity. She continued to have no movement in her right lower extremity and almost absent sensation in her left lower extremity. She also reported persistent saddle anesthesia and urinary retention.

## Discussion

Ventral spinal cord herniation can present in many ways including back and chest pain secondary to radiculopathy, and progressive myelopathy [3]. This is the first reported case of ventral spinal cord herniation causing diffuse SAH and intradural hematoma. In this case, the patient presented with chest pain which is nonspecific for spinal cord herniation, and other diagnoses were considered first including angina pectoris, pleurisy, gastrointestinal reflux disease, myalgia, and aortic dissection, and disorders of the esophagus and mediastinum. This, along with her lack of neurologic symptoms at the time of her first hospital visit is the reason why spinal imaging was not initially performed. When the patient arrived at the second hospital, she was paretic, which is why she was transferred to a third hospital for an emergent MRI. She did not become plegic until immediately before spinal angiography and surgical decompression.

Once the appropriate spinal imaging was obtained, the findings of cranial and cervical spine SAH as well as a T4 intradural hematoma prompted a spinal angiogram to look for a presumed underlying vascular malformation. The negative spinal angiogram led to the emergency decompression and exploration gave mass effect and unknown diagnosis. Operative findings confirmed that the T4 lesion was an intradural hematoma and that the ventral dural defect was causing spinal cord herniation. The cause of the dural defect remains unknown but may have been related to an MVA that the patient experienced 11 months prior. However, no good evidence exists regarding the etiology of dural defects in spinal cord herniation [11]. 

In addition, the cause of the acute cranial and cervical spine SAH (SSAH) and intradural hematoma in this case also remains unknown, especially given the absence of recent trauma or evidence of a ruptured aneurysm. SSAH is an uncommon cause of spinal cord or nerve root compression [12]. In fact, SSAH accounts for less than one percent of all cases of SAH [13,14]. The most common cause of SSAH is intracranial SAH, however, in this patient the cranial hemorrhage most likely originated from the spine. Less common causes of SSAH include trauma from lumbar punctures and underlying vascular malformations, such as arteriovenous malformations (AVMs), cavernomas, and spinal artery aneurysms [12]. On imaging, SSAH usually appears as the layering of blood in the CSF space (known as the sedimentation sign) or, less commonly, as a non-enhancing intradural-extramedullary clot causing mass effect on the spinal cord and/or nerve roots. The former is pathognomonic for SSAH [12]. One proposed theory among the authors as to why this patient developed SSAH is that progressive herniation of the cord over time ultimately led to the rupture of small (e.g., pial) vessels surrounding the spinal cord, causing SAH cranial to the site of herniation (i.e., in cervical spine and brain).

Of note, approximately one-third of patients with SSAH present with headache, meningismus, nausea, vomiting, seizure, sensorimotor deficits, and altered mental status, which this patient did not present with [12]. She did, however, present with acute onset back pain, motor paralysis, and sensory deficits, which are known spinal symptoms of SSAH [15-17].

Two major limitations of this case report are the lack of MR angiogram of the spine and, at the time of publication, no follow-up diagnostic angiography to identify the presence of micro-AVMs.

## Conclusions

Though ventral spinal cord herniation is well reported in the literature, this is the first reported case of spinal cord herniation-induced intradural hematoma and SAH. In addition, among the known causes of SSAH, spinal cord herniation has never been reported. The authors propose that sudden onset myelopathy and MRI findings consistent with spinal hematoma should raise concern for the possibility of ventral spinal cord herniation.

## References

[1] Alkan O, Kizilkilic O, Karakurum Goksel B, Yildirim T, Birol Sarica F (2008). Ventral thoracic spinal cord herniation: a commonly misdiagnosed and treatable cause of myelopathy. Neuroradiol J.

[2] Wortzman G, Tasker RR, Rewcastle NB, Richardson JC, Pearson FG (1974). Spontaneous incarcerated herniation of the spinal cord into a vertebral body: a unique cause of paraplegia. Case report. J Neurosurg.

[3] Senturk S, Guzel A, Guzel E (2008). Atypical clinical presentation of idiophatic thoracic spinal cord herniation. Spine (Phila Pa 1976).

[4] Eguchi T, Yokota H, Nikaido Y, Nobayashi M, Nishioka T (2001). Spontaneous thoracic spinal cord herniation--case report. Neurol Med Chir (Tokyo).

[5] Saito T, Anamizu Y, Nakamura K, Seichi A (2004). Case of idiopathic thoracic spinal cord herniation with a chronic history: a case report and review of the literature. J Orthop Sci.

[6] Borges LF, Zervas NT, Lehrich JR (1995). Idiopathic spinal cord herniation: a treatable cause of the Brown-Sequard syndrome--case report. Neurosurgery.

[7] Isu T, Iizuka T, Iwasaki Y, Nagashima M, Akino M, Abe H (1991). Spinal cord herniation associated with an intradural spinal arachnoid cyst diagnosed by magnetic resonance imaging. Neurosurgery.

[8] Kumar R, Taha J, Greiner AL (1995). Herniation of the spinal cord. Case report. J Neurosurg.

[9] Sioutos P, Arbit E, Tsairis P, Gargan R (1996). Spontaneous thoracic spinal cord herniation. A case report. Spine (Phila Pa 1976).

[10] Uchino A, Kato A, Momozaki N, Yukitake M, Kudo S (1997). Spinal cord herniation: report of two cases and review of the literature. Eur Radiol.

[11] Tyagi G, A R P, Bhat DI, Rao MB, Devi BI (2019). Duplication of ventral dura as a cause of ventral herniation of spinal cord—a report of two cases and review of the literature. World Neurosurg.

[12] Pierce JL, Donahue JH, Nacey NC (2018). Spinal hematomas: what a radiologist needs to know. Radiographics.

[13] Gonzalez LF, Zabramski JM, Tabrizi P, Wallace RC, Massand MG, Spetzler RF (2005). Spontaneous spinal subarachnoid hemorrhage secondary to spinal aneurysms: diagnosis and treatment paradigm. Neurosurgery.

[14] Moore JM, Jithoo R, Hwang P (2015). Idiopathic spinal subarachnoid hemorrhage: a case report and review of the literature. Global Spine J.

[15] Kreppel D, Antoniadis G, Seeling W (2003). Spinal hematoma: a literature survey with meta-analysis of 613 patients. Neurosurg Rev.

[16] Germans MR, Coert BA, Majoie CB (2015). Yield of spinal imaging in nonaneurysmal, nonperimesencephalic subarachnoid hemorrhage. Neurology.

[17] Sather MD, Gibson MD, Treves JS (2007). Spinal subarachnoid hematoma resulting from lumbar myelography. AJNR Am J Neuroradiol.

